# The association between oxcarbazepine-induced maculopapular eruption and HLA-B alleles in a Northern Han Chinese population

**DOI:** 10.1186/1471-2377-13-75

**Published:** 2013-07-08

**Authors:** Yu-Dan Lv, Fu-Li Min, Wei-Ping Liao, Na He, Tao Zeng, Di-Hui Ma, Yi-Wu Shi

**Affiliations:** 1 Department of Neurology, The First Affiliated Hospital of Jilin University, 71 Xinmin Street, Chang Chun, P. R. China; 2 Institute of Neuroscience and Second Affiliated Hospital of Guangzhou Medical University, Guangzhou 510260, China

**Keywords:** Mild maculopapular eruption, HLA-B* 1502, Northern Han Chinese, DNA genotyping

## Abstract

**Background:**

We investigated the association between oxcarbazepine (OXC)-induced maculopapular eruption (MPE) and HLA-B alleles in a northern Han Chinese population, and conducted an analysis of clinical risk factors for OXC-MPE.

**Methods:**

Forty-two northern Han Chinese patients who had been treated with OXC in Changchun, China were genotyped. Among them were 14 cases with OXC-induced MPE; the remaining 28 were OXC-tolerant. The HLA-B allele frequencies of the normal control group were found in the Allele Frequency Net Database. Polymerase chain reaction-sequence specific primer( PCR-SSP )was used for HLA-B*1502 testing and direct sequencing for four-digit genotype determination.

**Results:**

Four-digit allele sequencing showed that there was no statistically significant difference in the frequency of the HLA-B*1502 allele between the OXC-MPE and OXC-tolerant controls (3.6% versus 7.5%, OR = 0.38, 95% CI = 0.04–3.40, P = 0.65), as well as between OXC-MPE and normal controls (3.6% versus 2.4%, OR = 1.54, 95% CI = 0.20–11.73, P = 0.49). However, a significant difference in the frequency of HLA-B*3802 alleles was found between the MPE group and normal controls (10.7% versus 1.9%, OR = 6.329, 95% CI = 1.783-22.460, P = 0.018). There was no significant difference in terms of age, gender, or final OXC dose between the OXC-MPE and OXC-tolerant groups.

**Conclusions:**

There was no significant association between OXC-MPE and HLA-B*1502 in the northern Han Chinese population in our study. Instead, HLA-B*3802 was found to be a potential risk factor for OXC-MPE.

## Background

The new antiepileptic drug (AED) oxcarbazepine (OXC) is a 10-keto analog of carbamazepine (CBZ). The clinical effectiveness of OXC is similar to that of CBZ, but with fewer adverse drug reactions (ADRs), which is attributed to their different metabolic pathways [1]. A common side effect associated with AED use is rash, and has been the leading cause of withdrawal from some AED trials [2-4]. Recently it was reported that the incidence of OXC-related skin rashes is 3-8% in Norwegian [5,6] patients.

Cutaneous adverse drug reactions (cADRs) induced by AEDs range from mild maculopapular eruption (MPE) and hypersensitivity syndrome, to the more severe Stevens-Johnson syndrome (SJS) and toxic epidermal necrolysis (TEN). Among these cADRs, MPE is the most common [7], and is generally considered an initial form of the other, more severe, cADRs.

The pathogenesis of AED-induced cADRs is complex—several studies have indicated that T-cell-mediated allergic reactions might be associated with them [8], but genetic susceptibility and the exact pathogenesis are not fully understood and require further investigation. Recent studies have reported that the HLA-B*1502 allele is strongly associated with a dramatically increased risk of CBZ-induced SJS/TEN, but not MPE, among Han Chinese living in Taiwan and Hong Kong [9,10], that is, in southern China.

Owing to the structural similarity between OXC and CBZ, several studies of genetic susceptibility to OXC have been performed and an association between HLA-B*1502 and OXC-cADRs has been reported. These included studies that suggested that HLA-B*1502 may be associated with OXC-induced SJS/TEN [11,12]. Other studies such as Zhou D [13]reported that the HLA-B*1502 allele may contribute to genetic susceptibility to OXC-induced MPE in the Chinese Han population. However, there are also studies which found no significant association between HLA-B* 1502 and OXC-MPE in southern Han Chinese people [14]. Because of the inconsistencies among these results and the fact that there has been no study conducted to test the association between HLA-B*1502 and OXC-MPE in the northern Han Chinese population specifically, we carried out the following retrospective study. Herein we report our investigations into the association between OXC-induced MPE and HLA-B alleles. We also analyzed the clinical risk factors for OXC-induced MPE in a northern Han Chinese population.

## Methods

### Patients

All of the enrolled subjects (patients and normal controls) were of Northern Han Chinese ethnicity. Patients were from towns in Jilin Province (Hua Dian, Pan Shi, Tao Nan, Jiao He, and Shu Lan). The Ethics Committee of Jilin University at Changchun, and the Ethics Committee of Second Affiliated Hospital of Guangzhou Medical University at Guangzhou approved this study. All of the study participants provided written informed consent.

During the years 2010–2012, 14 cases that fulfilled the diagnostic criteria for OXC-induced MPE (OXC-MPE) were identified at the Department of Neurology, First Hospital of Jilin University, Changchun, China. MPE was defined as erythematous exanthema without blistering or postulation. The attribution of MPE to OXC treatment was determined jointly by the treating epileptologist and dermatologist.

We enrolled 28 subjects who had been administered OXC for more than 3 months but had not developed any cADRs (OXC-tolerant). In addition, for the normal control group we used the HLA-B allele frequencies reported in the Allele Frequency Net Database. Two control groups were used in this study, including 618 persons in Beijing, Shijiazhuang, Tianjin and 105 persons in the northern Han population (http://www.allelefrequencies.net).

Data collected from the patients’ records included: age, gender, initial and final dose of OXC, and latency to MPE (starting from the initial dose).

### Method

DNA was extracted from peripheral blood using a QIARamp blood mini kit (Qiagen, Hilden, Germany). The presence of HLA-B*1502 was examined via polymerase chain reaction with sequence-specific primers as in previous reports [14,15]. Sequencing for four-digit genotype determination was performed for all of the OXC-MPE and OXC-tolerant patients on an ABI 3730 sequencer (Applied Biosystems, Foster City, CA, USA). The forward and reverse primer sequences were Bin1-TAF (5'-TGGCGGGGGCGCAGGACCTGA-3') or Bin1-CGF (5'-TCGGGGGCGCAGGACCCGG-3') and Bin3-R (5'-CGGAGGCCATCCCCGGCGACCTAT-3'). Four-digit alleles of HLA-B in all patients were determined by sequence alignment using the immunogenetics/human leukocyte antigen (IMGT/HLA) database (http://www.ebi.ac.uk/imgt/hla) and the software vector NTI 6.0 (InforMax, Gaithersburg, MD, USA).

### Statistical analysis

Student’s *t*-test for independent samples was used to determine the significance of differences in mean age and dosage between the OXC-MPE and the OXC-tolerant groups. The chi-squared test and Fisher’s exact test were carried out to analyze the association between OXC-MPE and HLA-B alleles. To reduce bias in estimating the odds ratio (OR), whenever a zero-count field was encountered, 0.5 was added to all the fields in the 2×2 table [16]. The *P*-value from continuity correction (n ≥ 40 but 1 ≤ T ≤5) or Fisher’s exact test (n < 40 or T < 1), as well as estimated ORs and 95% exact confidence intervals (CIs), were established. *P*-values less than 0.05 (two-sided) were considered statistically significant. All analyses were performed using SPSS version 16.0 software (SPSS, Chicago, IL, USA).

## Results

Fourteen patients who had been diagnosed with OXC-induced MPE (9 females, 5 males; mean age 34.43 ± 12.10 y) and 28 patients who received OXC for at least 3 months without any evidence of adverse drug reactions (17 females, 11 males; mean age 34.04 ± 12.85 y) were enrolled as cases (OXC-MPE) and tolerant controls (OXC-tolerant), respectively. For the normal control group, we used the Allele Frequency Net Database (http://www.allelefrequencies.net, see Table 1).

**Table 1:** Clinical characteristics and genotypes in the 14 patients with OXC-induced MPE and in the 28 OXC-tolerant controls

**OXC-induced MPE**								
**ID No.**	**Gender**	**Phenotype**	**Initial-dose (mg)**	**Final-dose (mg)**	**Latency (d)**	**Concurrent drug**	**HLA-B 1502**	**Four-digit allele**
1	M	MPE	300	600	12	N	Negative	HLA-B*4001/4001
2	F	MPE	300	600	11	N	Negative	HLA-B*1302/4601
3	F	MPE	300	450	16	N	Negative	HLA-B*1302/1302
4	M	MPE	300	600	9	N	Negative	HLA-B*1501/5101
5	F	MPE	300	450	15	N	Negative	HLA-B*1501/1527
6	M	MPE	300	600	12	N	Negative	HLA-B*1501/4403
7	F	MPE	300	900	16	N	Negative	HLA-B*1501/1542
8	M	MPE	300	600	11	N	Negative	HLA-B*4001/1558
9	F	MPE	300	900	17	N	Negative	HLA-B*3802/4403
10	F	MPE	300	450	8	N	Negative	HLA-B*5201/5301
11	F	MPE	300	600	10	N	Negative	HLA-B*5502/5601
12	F	MPE	300	750	14	N	positive	HLA-B*3802/1502
13	M	MPE	300	750	12	N	Negative	HLA-B*1301/1301
14	F	MPE	300	600	9	N	Negative	HLA-B*4402/3802
**OXC-tolerant**								
**ID No.**	**Gender**	**Phenotype**	**Initial-dose (mg)**	**Final-dose (mg)**	**Latency (d)**	**Concurrent drug**	**HLA-B 1502**	**Four-digit allele**
15	F	Tolerant	300	900	N	N	Negative	HLA-B*4801/3531
16	F	Tolerant	300	600	N	N	Negative	HLA-B*4403/4804
17	M	Tolerant	300	600	N	N	Negative	HLA-B*4001/5501
18	M	Tolerant	300	900	N	N	Negative	HLA-B*3802/4403
19	F	Tolerant	300	900	N	N	Negative	HLA-B*4403/4403
20	M	Tolerant	300	600	N	N	Negative	HLA-B*3802/1302
21	F	Tolerant	300	600	N	N	Negative	HLA-B*4801/3531
22	M	Tolerant	300	450	N	N	Negative	HLA-B*4601/1511
23	M	Tolerant	300	600	N	N	Negative	HLA-B*1501/4001
24	F	Tolerant	300	450	N	N	Negative	HLA-B*1511/4801
25	F	Tolerant	300	600	N	N	Negative	HLA-B*4403/5604
26	F	Tolerant	300	900	N	N	Negative	HLA-B*1302/5801
27	M	Tolerant	300	900	N	N	Negative	HLA-B*1302/0705
28	F	Tolerant	300	600	N	N	Negative	HLA-B*3710/4701
29	F	Tolerant	300	600	N	N	Negative	HLA-B*1302/1315
30	F	Tolerant	300	600	N	N	Negative	HLA-B*1302/3901
31	F	Tolerant	300	900	N	N	Negative	HLA-B*5102/3508
32	M	Tolerant	300	900	N	N	Negative	HLA-B*5502/3801
33	F	Tolerant	300	450	N	N	Negative	HLA-B*1501/1513
34	F	Tolerant	300	600	N	N	Negative	HLA-B*1301/1301
35	M	Tolerant	300	600	N	N	Negative	HLA-B*2705/5101
36	M	Tolerant	300	450	N	N	Negative	HLA-B*4801/4801
37	F	Tolerant	300	600	N	N	positive	HLA-B*1502/4001
38	F	Tolerant	300	750	N	N	positive	HLA-B*5801/1502
39	F	Tolerant	300	900	N	N	Negative	HLA-B*4001/4006
40	M	Tolerant	300	600	N	N	positive	HLA-B*1502/5801
41	M	Tolerant	300	450	N	N	positive	HLA-B*4601/1502
42	F	Tolerant	300	900	N	N	positive	HLA-B*4601/1502

*OXC*, oxcarbazepine; *MPE*, maculopapular eruption; F, female; *M*, male.

The genotyping showed that HLA-B*1502 was present in only one of the 14 (7.1%) OXC-MPE patients, whereas 5 of the 28 (17.9%) OXC-tolerant controls carried this allele. The differences in the presence of HLA-B*1502 between the two groups was not statistically significant (OR = 0.35, 95% CI = 0.04–3.36, *P* = 0.64).

Four-digit allele sequencing showed that there was no statistically significant difference in the frequency of the HLA-B*1502 allele between the OXC-MPE and the OXC-tolerant control groups (3.6% versus 8.9%, OR = 0.38, 95% CI = 0.04–3.40, *P* = 0.65), as well as between the OXC-MPE group and the normal control group (3.6% versus 2.4%, OR = 1.54, 95% CI = 0.20–11.73, *P* = 0.49). Moreover, there were no significant differences between the OXC-MPE and the OXC-tolerant groups with regard to the frequencies of other HLA-B alleles. However, when the general northern Han population was used as a normal control, the frequency of the HLA-B*3802 allele was found to be significantly higher in the OXC-MPE group (10.7% versus 1.9%, OR = 6.329, 95% CI = 1.783-22.460, *P* = 0.018; Table 2).

**Table 2:** Frequencies of HLA-B alleles and their associations with OXC-induced MPE

	**Frequency**			**MPE cases compared with OXC-tolerant controls**		**MPE cases compared with normal population controls**	
**Alleles**	**MPE **^**a**^	**OXC-tolerant controls **^**b**^	**Population controls **^**c,d**^	**OR (95% CI)**	***P***	**OR (95% CI)**	***P***
0705	0/28 (0)	1/56 (1.8)	4/1236 ^c^ (0.4)	0.65 (0.03–16.45)	1.00	4.81 (0.25–91.38)	1.00
1301	2/28 (7.1)	2/56 (3.6)	44/1236 ^c^ (3.6)	2.08 (0.28–15.58)	0.86	2.08 (0.48–9.06)	0.62
1302	3/28 (10.7)	5/56 (8.9)	69/1236 ^c^ (5.5)	1.22 (0.27–5.54)	1.00	2.03 (0.60–6.89)	0.46
1315	0/28 (0)	1/56 (1.8)	–	0.65 (0.03–16.45)	1.00	–	–
1501	4/28 (14.3)	2/56 (3.6)	86/1236 ^c^ (7.0)	4.50 (0.77–26.27)	0.18	2.23 (0.76–6.57)	0.26
1502	1/28 (3.6)	5/56 (8.9)	29/1236 ^c^ (2.4)	0.38 (0.04–3.40)	0.65	1.54 (0.20–11.73)	0.49
1511	0/28 (0)	2/56 (3.6)	21/1236 ^c^ ( 1.7)	0.38 (0.02–8.24)	0.55	0.99 (0.06–16.78)	1.00
1513	0/28 (0)	1/56 (1.8)	1/1236 ^c^ (0.1)	0.65 (0.03–16.45)	1.00	14.45 (0.58–362.44)	1.00
1527	1/28 (3.6)	0/56 (0)	2/1236 ^c^ (0.2)	6.16 (0.24–156.27)	0.33	22.85 (2.01–259.72)	0.07
1542	1/28 (3.6)	0/56 (0)	0/210 ^d^ (0)	6.16 (0.24–156.27)	0.33	22.96 (0.91–577.76)	0.12
1558	1/28 (3.6)	0/56 (0)	2/1236 ^c^ (0.2)	6.16 (0.24–156.27)	0.33	22.85 (2.01–259.72)	0.07
2705	0/28 (0)	1/56 (1.8)	6/1236 ^c^ (0.5)	0.65 (0.03–16.45)	1.00	3.32 (0.18–60.38)	1.00
3508	0/28 (0)	1/56 (1.8)	1/1236 ^c^ (0.1)	0.65 (0.03–16.45)	1.00	14.45 (0.58–362.44)	1.00
3531	0/28 (0)	2/56 (3.6)	0/210 ^d^ (0)	0.38 (0.02–8.24)	0.55	–	–
3710	0/28 (0)	1/56 (1.8)	–	0.65 (0.03–16.45)	1.00	–	–
3801	0/28 (0)	1/56 (1.8)	8/1236 ^c^ (0.7)	0.65 (0.03–16.45)	1.00	2.54 (0.14–45.00)	1.00
3802	3/28 (10.7)	2/56 (3.6)	23/1236 ^c^ (1.9)	3.24 (0.51–20.63)	0.42	6.329 (1.783–22.460)	0.018 ^e^
3901	0/28 (0)	1/56 (1.8)	23/1236 ^c^ (1.9)	0.65 (0.03–16.45)	1.00	0.91 (0.05–15.29)	1.00
4001	3/28 (10.7)	4/56 (7.1)	100/1236 ^c^ (8.1)	1.56 (0.32–7.51)	0.89	1.36 (0.41–4.59)	0.88
4006	0/28 (0)	1/56 (1.8)	39/1236 ^c^ (3.2)	0.65 (0.03–16.45)	1.00	0.53 (0.03–8.87)	1.00
4402	1/28 (3.6)	0/56 (0)	11/1236 ^c^ (0.9)	6.16 (0.24–156.27)	0.33	4.13 (0.51–33.09)	0.24
4403	2/28 (7.1)	5/56 (8.9)	32/1236 ^c^ (2.6)	0.79 (0.14–4.32)	1.00	2.89 (0.66–12.72)	0.17
4601	1/28 (3.6)	3/56 (5.4)	118/1236 ^c^ (9.6)	0.65 (0.07–6.59)	1.00	0.35 (0.05–2.61)	0.46
4701	0/28 (0)	1/56 (1.8)	0/210 ^d^ (0)	0.65 (0.03–16.45)	1.00	–	–
4801	0/28 (0)	5/56 (8.9)	24/1236 ^c^ (2)	0.16 (0.01–3.08)	0.25	0.87 (0.05–14.63)	1.00
4804	0/28 (0)	1/56 (1.8)	0/210 ^d^ (0)	0.65 (0.03–16.45)	1.00	–	–
5101	1/28 (3.6)	1/56 (1.8)	66/1236 ^c^ (5.4)	2.04 (0.12–33.83)	1.00	0.66 (0.09–4.91)	1.00
5102	0/28 (0)	1/56 (1.8)	6/1236 ^c^ (0.5)	0.65 (0.03–16.45)	1.00	3.32 (0.18–60.38)	1.00
5201	1/28 (3.6)	0/56 (0)	21/1236 ^c^ (1.9)	6.16 (0.24–156.27)	0.33	2.14 (0.28–16.51)	0.39
5301	1/28 (3.6)	0/56 (0)	0/210 ^d^ (0)	6.16 (0.24–156.27)	0.33	22.96 (0.91–577.76)	0.12
5501	0/28 (0)	1/56 (1.8)	7/1236 ^c^ (0.6)	0.65 (0.03–16.45)	1.00	2.88 (0.16–51.58)	1.00
5502	1/28 (3.6)	1/56 (1.8)	35/1236 ^c^ (2.9)	2.04 (0.12–33.83)	1.00	1.27 (0.17–9.62)	0.56
5601	1/28 (3.6)	0/56 (0)	2/1236 ^c^ (0.2)	6.16 (0.24–156.27)	0.33	22.85 (2.01–259.72)	0.07
5604	0/28 (0)	1/56 (2.5)	1/1236 ^c^ (0.1)	0.65 (0.03–16.45)	1.00	14.45 (0.58–362.44)	1.00
5801	0/28 (0)	3/56 (5.3)	74/1236 ^c^ (6)	0.27 (0.01–5.38)	0.55	0.27 (0.02–4.53 )	0.35

^a^ 2n = 28.

^b^ 2n = 56.

^c^ 2n = 1236, from a China Beijing Shijiazhuang Tianjin Han population of 618 persons (http://www.allelefrequencies.net).

^d^ 2n = 210, from a China North Han population of 105 persons (http://www.allelefrequencies.net). Note: The frequency of these alleles has not been reported by the former.

OXC, oxcarbazepine; MPE, maculopapular eruption; OR, odds ratio; CI: confidence interval.

^e^*P* < 0.05 (two-sided) was statistically significant.

–, no data available.

There was no significant difference between the patients with or without OXC-MEP in terms of age (34.43 ± 12.10 versus 34.04 ± 12.85, *P* = 0.93), gender (male/female: 5/9 versus 11/17, OR = 0.86, 95% CI = 0.23-3.25, *P* = 0.82), or final dose (632.14 ± 146.24 mg/d versus 675.00 ± 170.78 mg/d, *P* = 0.43; Table 1).

## Discussion

In the present study we found no association between OXC-induced MPE and HLA-B*1502 in our northern Han Chinese patients. Our result is consistent with previous studies of southern Han Chinese [14], but different from another study showing a significant association between OXC-induced MPE and HLA-B*1502 in central Han Chinese [7]. These discrepancies could mainly be due to differences in the frequency of HLA-B*1502 in different populations. Of note, in each of these studies the sample size was small, which limited the power of the statistical analyses.

Among Han Chinese living in Taiwan and Hong Kong (southern China), HLA-B*1502 was strongly associated with a dramatically increased risk of CBZ-induced SJS/TEN, but was not associated with MPE [10]. Recently, three OXC-induced SJS/TEN cases reported from Taiwan were positive for HLA-B*1502 [17], and there was one case of OXC-induced SJS positive for HLA-B*1502 in India [18]. This suggests a very strong association between OXC-induced SJS/TEN and the HLA-B*1502 allele. In our study, we found that the HLA-B*1502 allele was only present in 7.1% of OXC-induced MPE patients in this northern Han Chinese population.

This may be due to the inflammatory immune reactions of MPE different from SJS/TEN. CD4 ^+^ T cells are the major cell type found in the skin lesions of MPE [19], whereas CD8 ^+^ T-cell-mediated cytotoxic responses appear to be the major event in SJS/TEN [20].

OXC is a new AED which has shown clinical effectiveness similar to that of CBZ but with fewer adverse drug reactions; therefore OXC is now frequently used as a substitute for CBZ. Rash is a common side effect of AED therapy and is the leading cause of withdrawal from AED prescriptions. Some of these rashes can be ameliorated by early-stage therapy with additional management to prevent them from developing into more severe cutaneous reactions that could be fatal, while discontinuing OXC due to skin eruption prevents the long-term use of what would otherwise be an effective drug, and increases the suffering of patients. Hence, identifying the possible risk factors for cADRs will contribute to their prevention and the safe use of OXC.

We therefore compared the frequencies of HLA-B alleles other than HLA-B*1502 between the OXC-MPE and the OXC-tolerant groups. We found an association between the HLA-B*3802 allele and OXC-induced MPE in the northern Han Chinese population we studied; the frequency of the HLA-B*3802 allele was significantly higher in the OXC-MPE group. However, it should be noted that the general northern Han population was used as a control group. When OXC-tolerant subjects were used as a control, there was no statistically significant difference in the frequency of the HLA-B*3802 allele between the OXC-MPE and the OXC-tolerant control groups. This discrepancy may be because this is a small sample study, and therefore the statistical power to detect any significant difference between cases and controls was limited. Whether the HLA alleles identified in this exploratory study would be associated with the development of OXC-induced MPE in a larger sample needs to be investigated.

Hung et al. [9] found that MPE induced by CBZ was associated with single nucleotide polymorphisms (SNPs) in the HLA-E region and the nearby allele HLA-A*3101, but hypersensitivity syndrome was associated with SNPs in the motilin gene located terminal to the major histocompatibility complex (MHC) class II genes [8]. Therefore, the association between OXC-induced skin rashes and other HLA alleles beyond HLA-B should be explored in future research.

Our analysis of potential risk factors for OXC-induced MPE in the present study showed that neither age, gender, nor final dose of OXC were associated with incidence of the skin rash, which is consistent with previous studies in Korea [21]. However, other related studies showed that several clinical variables affected skin reactions, including gender, hormones, the drug titration schedule, and co-medication [22]. Among these variables, a high starting dose and rapid dose escalation were identified as risk factors, especially for the AED lamotrigine, and particularly when administered concurrent with valproate [23]. In addition, it has been reported that a reaction to one AED may induce hypersensitivity to a previously tolerated AED, and a previous AED-related skin reaction appears to be a considerable risk factor for occurrence of another rash [24]. The differences between our study and those cited above may be because all of the patients in our study were given monotherapy, treated with the same initial dosage, and the small sample size.

## Conclusion

In conclusion, we found no significant association between OXC-induced MPE and the HLA-B*1502 allele in the northern Han Chinese patients we studied. Instead, in this Han Chinese population HLA-B*3802 was identified as a potential risk factor for OXC-MPE. Furthermore, no association was found between OXC-MPE and age, gender, or dosage. It should be noted that this is a small-sample study, which limited the statistical power to detect any significant difference. Therefore, our results need to be further confirmed by a future study with a larger sample size.

## Abbreviations

AED: Antiepileptic drug; cADR: Cutaneous adverse drug reaction; CBZ: Carbamazepine; CI: Confidence interval; MPE: Mild maculopapular eruption; OR: Odds ratio; OXC: Oxcarbazepine; PCR: Polymerase chain reaction; SJS: Stevens-Johnson syndrome; TEN: Toxic epidermal necrolysis.

## Competing interests

The authors declared no potential conflicts of interest with respect to the research, authorship, finance and/or publication of this article.

## Authors' contributions

All authors have read and approved the final manuscript. YDL and FLM contributed equally to this work, as First Authors. YDL contributed to the conception, design, acquisition of data, and writing. FL M contributed to analysis, interpretation of data, and writing. D-HM and YWS contributed equally as corresponding author. DHM contributed to giving final approval of the version to be published; Y-WS contributed to manuscript revision, general supervision of the research group, and giving final approval of the version to be published. WPL participated in the design of the study. NH performed the statistical analysis and revising. TZ participated in the sequence alignment.

## Pre-publication history

The pre-publication history for this paper can be accessed here:

http://www.biomedcentral.com/1471-2377/13/75/prepub

## References

[B1] Rambeck B Specht U Wolf P Pharmacokinetic interactions of the new antiepileptic drugs Clin Pharmacokinet 1996 31 309 24 10.2165/00003088-199631040-00006 8896946

[B2] Chadwick D Shaw MBM Foy P Rawlins MD Turnbull DM Serum anticonvulsant concentrations and the risk of drug induced skin eruptions J Neurol Neurosurg Psychiatry 1984 47 6 642 4 10.1136/jnnp.47.6.642 6234378 PMC1027864

[B3] Kramlinger KG Phillips KA Post RM Rash complicating carbamazepine therapy J ClinPsychopharmacol 1994 14 6 408 13 7884021

[B4] Brodie MJ Richens A Yuen AW Double-blind comparison of lamotrigine and carbamazepine in newly diagnosed epilepsy Lancet 1995 345 8948 476 9 10.1016/S0140-6736(95)90581-2 7710545

[B5] Hirsch LJ Arif H Nahm EA Buchsbaum R Resor SR Bazil CW Cross-sensitivity of skin rashes with antiepileptic drug use Neurology 2008 71 19 1527 34 10.1212/01.wnl.0000334295.50403.4c 18981374

[B6] Alvestad S Lydersen S Brodtkorb E Rash from antiepileptic drugs: influence by gender, age, and learning disability Epilepsia 2007 48 7 1360 5 10.1111/j.1528-1167.2007.01109.x 17484761

[B7] Hu FY Wu XT An DM Yan B Stefan H Zhou D Pilot association study of oxcarbazepine-induced mild cutaneous adverse reactions with HLA-B*1502 allele in Chinese Han population Seizure 2011 20 2 160 2 10.1016/j.seizure.2010.11.014 21169036

[B8] Zanni MP Schnyder B von Greyerz S Pichler WJ Involvement of T cells in drug-induced allergies Trends Pharmacol Sci 1998 19 8 308 10 10.1016/S0165-6147(98)01227-9 9745357

[B9] Hung SI Chung WH Jee SH Genetic susceptibility to carbamazepine-induced cutaneous adverse drug reactions Pharmacogenet Genom 2006 16 4 297 306 10.1097/01.fpc.0000199500.46842.4a 16538176

[B10] Man CB Kwan P Baum L Association between HLA-B*1502 allele and antiepileptic drug-induced cutaneous reactions Epilepsia 2007 48 4 1015 8 17509004 10.1111/j.1528-1167.2007.01022.x

[B11] Shankarkumar U Shah KN Ghosh K HLA B*1502 allele association with oxcarbamazepine-induced skin reactions in epilepsy patient from India Epilepsia 2009 50 7 1837 8 10.1111/j.1528-1167.2009.02096.x 20831525

[B12] Chen YC Chu CY Hsiao CH Oxcarbazepine-induced Stevens-Johnson syndrome in a patient with HLA-B*1502 genotype J Eur Acad Dermatol Venereol 2009 23 6 702 3 10.1111/j.1468-3083.2008.02988.x 18785891

[B13] Wu XT Hu FY An DM Yan B Jiang X Zhou D Association between carbamazepine-induced cutaneous adverse drug reactions and the HLA-B*1502 allele among patients in central China Epilepsy Behav 2010 19 3 405 8 10.1016/j.yebeh.2010.08.007 20833111

[B14] He N Min FL Shi YW Guo J Liu XR Li BM Cutaneous reactions induced by oxcarbazepine in Southern Han Chinese: Incidence, features, risk factors and relation to HLA-B alleles Seizure 2012 21 8 614 8 10.1016/j.seizure.2012.06.014 22818943

[B15] Chung WH Hung SI Hong HS Hsih MS Yang LC Ho HC Medical genetics: a marker for Stevens Johnson syndrome Nature 2004 428 6982 486 10.1038/428486a 15057820

[B16] Svensson CK Cowen EW Gaspari AA Cutaneous drug reactions Pharmacol Rev 2001 53 3 357 79 11546834

[B17] Hung SI Chung WH Liu ZS Chen CH Hsih MS Hui RC Common risk allele in aromatic antiepileptic-drug induced Stevens–Johnson syndrome and toxic epidermal necrolysis in Han Chinese Pharmacogenomics 2010 11 3 349 56 10.2217/pgs.09.162 20235791

[B18] Sharma SR Sharma N Yeolekar ME Oxcarbazepine-induced Stevens-Johnson syndrome: a case report Indian Dermatol Online J 2011 2 1 13 15 10.4103/2229-5178.79861 23130207 PMC3481788

[B19] Naisbitt DJ Britschgi M Wong G Farrell J Depta JP Chadwick DW Hypersensitivity reactions to carbamazepine: characterization of the specificity, phenotype, and cytokine profile of drug-specific T cell clones Mol Pharmacol 2003 63 3 732 741 10.1124/mol.63.3.732 12606784

[B20] Nassif A Bensussan A Boumsell L Deniaud A Moslehi H Wolkenstein P Toxic epidermal necrolysis: effector cells are drug-specific cytotoxic T cells J Allergy Clin Immunol 2004 114 5 1209 1215 10.1016/j.jaci.2004.07.047 15536433

[B21] Park KJ Kim JR Joo EY Seo DW Hong SB Drug Interaction and Pharmacokinetic Modeling of Oxcarbazepine in Korean Patients With Epilepsy Clin Neuropharmacol 2012 35 1 40 4 10.1097/WNF.0b013e31824150a5 22246398

[B22] Zaccara G Franciotta D Perucca E Idiosyncratic adverse reactions to antiepileptic drugs Epilepsia 2007 48 7 1223 44 10.1111/j.1528-1167.2007.01041.x 17386054

[B23] Arif H Buchsbaum R Weintraub D Koyfman S Salas-Humara C Bazil CW Comparison and predictors of rash associated with 15 antiepileptic drugs Neurology 2007 68 20 1701 9 10.1212/01.wnl.0000261917.83337.db 17502552

[B24] Klassen BD Sadler RM Induction of hypersensitivity to a previously tolerated antiepileptic drug by a second antiepileptic drug Epilepsia 2001 42 3 433 5 11442165 10.1046/j.1528-1157.2001.33400.x

